# Complicating factors in the management of advanced Bouveret syndrome in frail and medically complex patients: Case report and discussion of pathophysiology

**DOI:** 10.1016/j.ijscr.2020.10.076

**Published:** 2020-10-22

**Authors:** Zachary Obinna Enumah, Evan G. Wong, Alistair J. Kent

**Affiliations:** a Johns Hopkins University School of Medicine, Department of Surgery, Baltimore, MD, USA; b McGill University, Department of Surgery, Montreal, Canada

**Keywords:** Gastric outlet obstruction, Biliary disease, Geriatrics, Cholecystostomy, Gallstone ileus, Enterolithiasis

## Abstract

**Introduction:**

Bouveret Syndrome is a rare but important variant of gallstone ileus with high potential for morbidity and mortality. Bouveret syndrome is a complication of gallstone disease resulting from chronic inflammation and subsequent fistulization between the gallbladder and duodenum or stomach with subsequent impaction of the stone in the proximal GI tract. Here we present a case in an elderly man with moderate medical comorbidities.

**Presentation of case:**

An elderly man presented to the hospital with symptoms of gastrointestinal obstruction. Upon further diagnostic work-up, he was noted to have a 5.8 cm gallstone impacted in his proximal GI tract and thus diagnosed with a rare variant of gallstone ileus—Bouveret syndrome.

**Discussion:**

The therapeutic goal in approaching Bouveret syndrome is removal of the stone and improvement in obstruction and cholangitis. This may be accomplished with surgery or endoscopic therapy—although this may be less effective. Bouveret syndrome may have high morbidity.

**Conclusion:**

Bouveret syndrome is a rare but potentially serious syndrome that should be managed accordingly. It should remain on the differential diagnosis of an elderly patient presenting with gastrointestinal obstructions, particularly if there is a history of gallstone disease and concern for proximal GI obstruction.

## Highlights

• Bouveret’s syndrome results from biliary stones from cholecysto-duodenal fistula.
• Gastric outlet obstruction from duodenal stones has high morbidity and mortality.
• Duodenal gallstones may grow over time, increasing risks of management.
• Delay in diagnosis and stone extraction may result in needing higher risk surgery.
• Tertiary referral of at-risk elderly or frail patients may improve outcomes.

## 1 Introduction

Bouveret syndrome is a rare sub-type of gallstone ileus whereby a stone becomes lodged in the duodenum or stomach—resulting in symptoms typical of gastric outlet obstruction. Diagnosis may be difficult as it may present with non-specific signs. Management is typically endoscopic or surgical in nature involving stone extraction with or without cholecystectomy and/or fistula takedown. Here we present a case of Bouveret syndrome in an elderly man with multiple medical comorbidities.

## 2 Case presentation

**Fig. 1:**
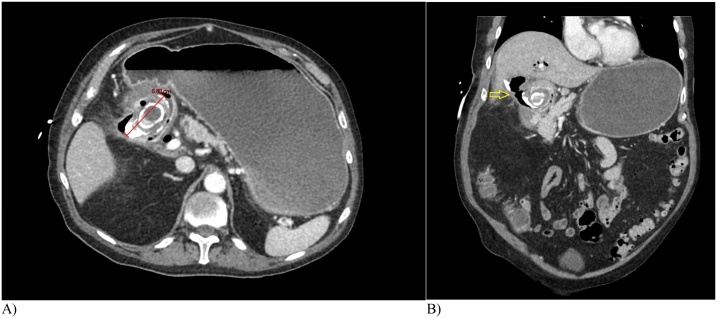
Computed Tomography Abdomen Axial and Coronal. A) 5.8 cm (red line) laminated and partially calcified stone is noted in the first and second portions of the duodenum with B) pneumobilia suggesting cholecysto-duodenal fistula (yellow arrow) and associated signs of gastric outlet obstruction.

An 81-year-old man with medical history significant for atrial fibrillation (on rivaroxaban), chronic kidney disease, hypertension, hyperlipidemia, glaucoma, gout and melanoma status post excision presented with cholecystitis that was managed with a cholecystostomy drain at another institution and symptoms resolved. Four months later he represents with several days of nausea, bilious vomiting, and abdominal pain. At presentation, he had normal bowel function with a last bowel movement approximately two days prior to admission. The patient was a former smoker and non-drug user without alcohol history. He had no significant family history recorded. On physical exam, he was afebrile, and his vital signs were notable for irregular heart rhythm, consistent with atrial fibrillation with rapid ventricular response (heart rate in the 130 s), and he demonstrated moderate right upper quadrant tenderness; his cholecystostomy tube remained in place with dark, thick bilious drainage and flushed easily. Laboratory work on admission showed white blood cell 10.9 K/cu mm. Liver enzymes were normal with aspartate amino transaminase of 15 U/L, alanine amino transaminase of 9 U/L, alkaline phosphatase of 78 U/L, and total bilirubin of 1.2 mg/dL. CT scan demonstrated pneumobilia, massive gastric distension, and an obstructing 5.8 cm calcified intraluminal mass in the first and second portion of the duodenum (Fig. 1).

The patient was placed on broad spectrum antibiotics to control early a potential development of cholangitis and a nasogastric tube was placed for gastric decompression. Heart rate control was managed with betablockers and calcium channel blockers. In preparation for surgery, his anticoagulation was held, and total parenteral nutrition was initiated.

**Fig. 2:**
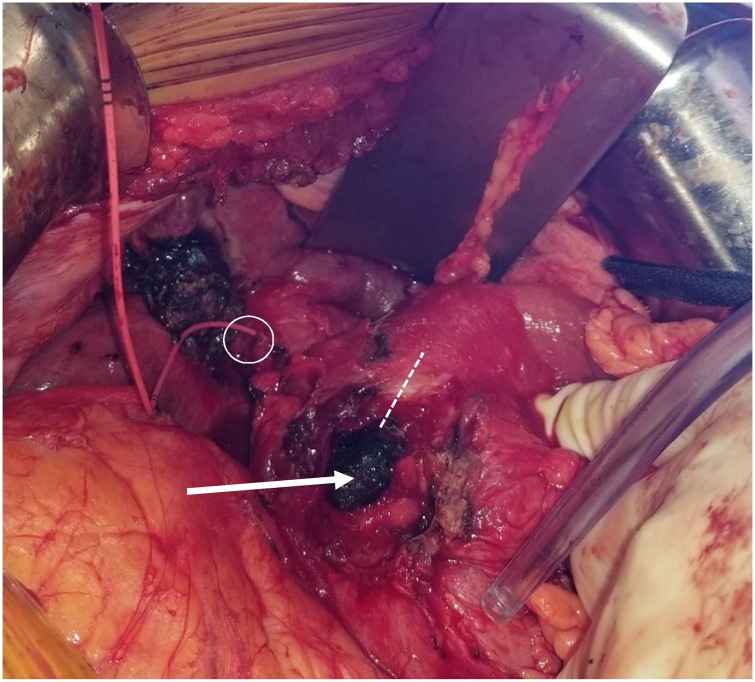
Intraoperative View and Gross Specimen – Exposure of impacted obstructing calculus (white arrow) in situ after fistula takedown. Pylorus (dashed line) incised to allow for stone extraction. Fogarty catheter placed through cystic (circle) duct stump into duodenum to enable identification of CBD and location of Ampula of Vater.

**Fig. 3:**
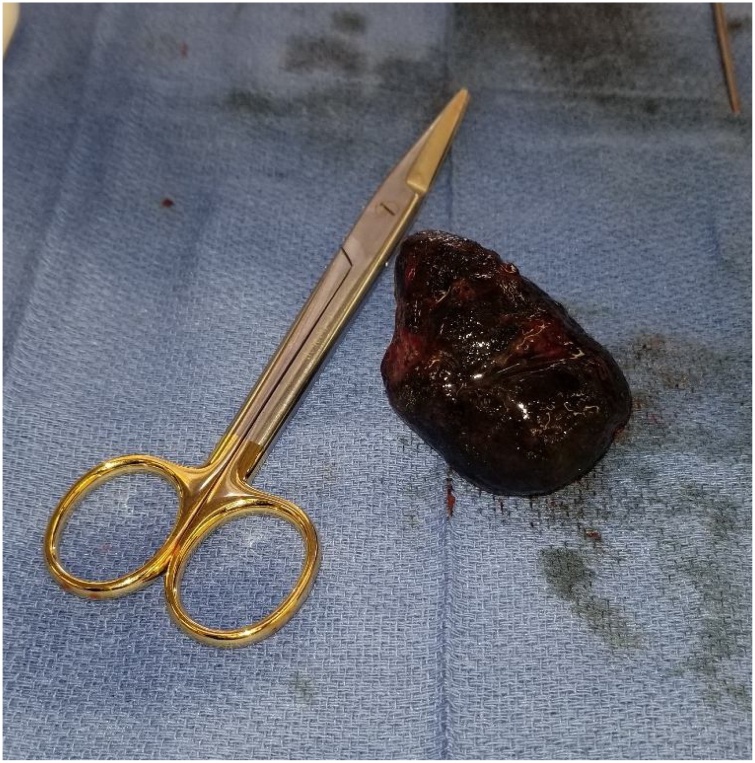
Calculus after removal with scissors for reference; tapered end of stone noted at site of obstruction suggesting growth of stone proximally after impaction.

The patient underwent exploration via a right subcostal incision. The location of the impacted stone was readily identified in the first and second portions of the duodenum (D1 and D2, respectively) which were severely inflamed up to and including the pylorus, with the gallbladder adherent to the anterolateral surface of D1 and D2. Several maneuvers including partial Kocherization, were utilized in attempt to mobilize the stone for extraction via gastrotomy; however due to the large size, location of the cholecysto-duodenal fistula, degree of impaction of the stone, and surrounding inflammation, this was not feasible. Cholecystectomy and cholecysto-duodenal fistula takedown were then performed, where it was noted that the stone was substantially (2–3x the diameter) of the fistula. This required extension of the defect over the anterolateral surface of D1 through the pylorus and a 6.2 × 4.2 × 3.5 cm stone was successfully removed (Fig. 2, Fig. 3). The edges of the fistula were freshened and reconstruction was accomplished with Roux-en-Y duodenojejunostomy and gastrojejunostomy tube placement.

The patient’s intraoperative course was additionally remarkable for hemodynamic instability, atrial fibrillation with rapid ventricular response and sepsis during manipulation of the liver and duodenum. Management required anti-arrhythimics, electrical cardioversion, multiple vasopressors, blood product transfusions and continuous intraoperative transoesophageal echocardiography. Blood loss was modest. He was taken to the intensive care unit post-operatively and he rapidly recovered. Vasoactive medications were weaned, and he was extubated on post-operative day 1. On post-operative day 6, he had an upper gastrointestinal (GI) contrast study without evidence of leak; jejunal tube feeds were initiated and tolerated well. TPN was discontinued. He had post-obstructive gastric emptying dysfunction and required decompression through the gastric port of his gastrojejunostomy tube for several days prior to successfully transitioning to a regular diet. He was discharged to a rehabilitation center on post-operative day 21. His gastrostomy tube was removed at a follow-up clinic visit.

## 3 Discussion

Gastrointestinal obstruction accounts for a significant percentage of admissions for abdominal pain, but among the many common causes of obstruction (e.g. hernia, malignancy, adhesive disease), gallstone ileus accounts for less than 5% [1]. When gallstone ileus does occur, the most common sites of obstruction are the ileum (60%), jejunum, (15%), stomach (15%), and colon (5%) [2]. Nevertheless, among patients over 65 years of age, gallstone ileus may actually account for up to 25% of non-strangulated small bowel obstructions [3]. This patient presented with pneumobilia and proximal GI obstruction with concerns for cholangitis given the stone’s location and constellation of signs and symptoms. This was due to the development of a fistula between the fundus of the gallbladder and the proximal duodenum from erosion of a large gallstone that subsequently became lodged above the level of the ampulla of Vater. His cystic duct had re-canalized after his previous cholecystitis (as evidenced by bilious cholecystostomy tube drainage) allowing for an anomalous open connection between the proximal GI tract and the common bile duct.

First described by Beaussier in the 1700s and later reported by Leon Bouveret in 1896, Bouveret syndrome is a rare sub-type of gallstone ileus whereby a stone becomes lodged in the duodenum or stomach—resulting in symptoms typical of gastric outlet obstruction [4]. Bouveret syndrome is a complication of gallstone disease resulting from chronic inflammation and subsequent fistulization between the gallbladder and duodenum or stomach with subsequent impaction of the stone in the proximal GI tract [5]. It accounts for less than 3% of cases of gallstone ileus [6]. A patient would typically present with subacute obstructive symptoms, though may have had chronic upper/right side abdominal pain or findings of gallstones [4,7]. Ultrasound findings are most often consistent with gallstones and pneumobilia; similarly, a single gallstone of 2.5 cm or greater is the most common cause of Bouveret syndrome [7,8]. Diagnosis is often made during standard workup for abdominal pain or proximal GI obstruction and may include abdominal radiography, fluoroscopy, computed tomography (CT) or endoscopy. Oral contrast filling the gallbladder can indicate a cholecysto-enteral fistula [8], a pathognomonic feature of the disease process secondary to erosion by the stone through the gallbladder wall into the GI tract.

The therapeutic goal in approaching Bouveret syndrome is relief of the obstruction, typically by the removal or fragmentation of the stone. As in other enterolithiases, a cholecysto-enteral fistula may be well tolerated in the absence of distal obstruction causing cholangitis. Initial attempts at management of Bouveret syndrome by endoscopic therapy should be accompanied with consideration of the risks of advanced endoscopic techniques in the setting of a hostile field, particularly including duodenal or intestinal perforation requiring subsequent surgical intervention [12,13]. Management is often surgical and includes gastrotomy, pyloromyotomy or enterotomy with or without cholecystectomy and fistula repair [[8], [9], [10], [11]].

The surgical approach may be conducted in one or two stages [14]. The one-stage procedure consists of enterolithotomy, cholecystectomy and repair of chole-enteric fistula, whereas two stage aims for enterolithotomy (e.g. via an isolated pyloromyotomy proximal to the fistula site) followed by optional interval cholecystectomy with fistula takedown [15]. The two-stage procedure is not always feasible as there may be severe inflammation and stone removal may require concurrent fistula takedown. However, if isolated stone removal is accomplished via endoscopic or surgical extraction and there is no distal obstruction, repair of the fistula is not obligatory.

The location of cholecysto-enteral fistulization directly impacts the presentation of the disease; Bouveret syndrome specifically requires a fistula in the stomach or duodenum and a stone large enough to obstruct this particular anatomical location of the duodenum. Fistulas from gallstone ileus appear to commonly be cholecysto-duodenal in nature [16]. We noted that in this case the relatively narrower second portion of the duodenum, adjacent to the bulk of the pancreas and peri-ampullary complex appeared to have blocked the passage of the stone which fistulized through the antero-lateral portion of distal D1. The stone was substantially larger (approximately 2–3x the diameter) than the fistula through which it passed from gallbladder to duodenum, and was contoured to the shape of the distended proximal duodenum with tapering at the site of obstruction suggesting that the stone continued to grow after becoming lodged (Fig. 3). We hypothesize this may represent a staged progression in the pathophysiology of Bouveret syndrome that has not been previously elaborated in the literature. In addition to the anatomic limitations of fistula location and degree of surrounding inflammation, stone growth might hypothetically explain some of the differentiation in previous reports of success regarding endoscopic management and perhaps, analogously, the success rates of stone extraction vs. resection for gallstone ileus [17,18]. This would suggest that gallstones lodged in the proximal duodenum by way of cholecysto-duodenal fistulization may be substantially easier to address by less invasive techniques early in their course before becoming impacted and necessitating the increased risk of surgical extraction or fistula takedown.

In this case, a relatively robust elderly patient with good nutritional status and moderate comorbidities (American Society of Anesthesiologists Score 3) who would have likely tolerated elective—or perhaps even urgent cholecystectomy earlier in his course—developed a severe, life threating, and time sensitive complication requiring invasive surgery. As demonstrated, manipulation of the infected biliary system during surgery may exacerbate sepsis from pre-existing cholangitis and require advanced anesthesia capabilities, especially in the setting of moderate or severe medical comorbidities. We chose to proceed directly with surgery predominately because of large stone size (associated with higher scope failure rates) and surrounding inflammation. We inspected to see if stone could be mobilized for extraction through a gastrotomy but it was completely fixed and non-mobile in the space between ampulla and pylorus even after partial kocherization so we proceeded with takedown of the gallbladder and extraction of the stone from the (enlarged) fistula track.

Regardless of the approach taken, Bouveret syndrome is a rare but potentially serious syndrome that should be managed accordingly. Diagnosis can be difficult due to the slow onset and lack of specific symptoms to differentiate from other biliary disease. Patients with known gallstones with persistent symptoms should be considered at risk for development of this uncommon but severely morbid condition; this is particularly relevant for patients considered poor candidates for cholecystectomy due to age, frailty, or medical complexity. Early referral of such patients to a tertiary facility with advanced anesthesia, critical care, and endoscopic capabilities in addition to surgeons with expertise in hepatobiliary disease could prevent or reduce the severity of devastating complications in these vulnerable populations.

This case report has been written in compliance with the SCARE Guidelines 2018 [19].

## Declaration of Competing Interest

No conflicts of interest.

## Funding

No funding obtained.

## Ethical approval

As this is a case study, ethical approval was not obtained as no identifiable patient information was shared or published in personally identifiable form. Appropriate consent was obtained directly from the patient, and a copy of the manuscript draft was provided to him/her/them.

## Consent

Consent was obtained from the patient for the publication of this case report. A written form can be made available by request of the journal. A copy of the manuscript draft was also shared with the patient.

## Author’s contribution

Conceptualization: ZOE, EGW, AJK.

Data curation: ZOE, EGW, AJK.

Formal Analysis: ZOE, EGW, AJK.

Funding Acquisition: ZOE.

Investigation: ZOE, EGW, AJK.

Methodology: ZOE, EGW, AJK.

Project Administration/Resources: ZOE, AJK.

Supervision: AJK.

Validation: N/A.

Visualization: N/A.

Original Draft: ZOE, EGW, AJK.

Review and Editing: ZOE, EGW, AJK.

## Registration of research studies

N/A.

## Guarantor

Alistair J Kent, MD MPH.

## Provenance and peer review

Not commissioned, externally peer-reviewed.
